# 3D Printed Microfluidic Spiral Separation Device for Continuous, Pulsation-Free and Controllable CHO Cell Retention

**DOI:** 10.3390/mi12091060

**Published:** 2021-08-31

**Authors:** Anton Enders, John-Alexander Preuss, Janina Bahnemann

**Affiliations:** 1 Institute of Technical Chemistry, Leibniz University Hannover, 30167 Hannover, Germany; enders@iftc.uni-hannover.de (A.E.); preuss@iftc.uni-hannover.de (J.-A.P.); 2 Cell Culture Technology, Faculty of Technology, Bielefeld University, 33615 Bielefeld, Germany

**Keywords:** microfluidics, 3D printing, inertial microfluidics, continuous cultivation, cell retention, CHO cells

## Abstract

The development of continuous bioprocesses—which require cell retention systems in order to enable longer cultivation durations—is a primary focus in the field of modern process development. The flow environment of microfluidic systems enables the granular manipulation of particles (to allow for greater focusing in specific channel regions), which in turn facilitates the development of small continuous cell separation systems. However, previously published systems did not allow for separation control. Additionally, the focusing effect of these systems requires constant, pulsation-free flow for optimal operation, which cannot be achieved using ordinary peristaltic pumps. As described in this paper, a 3D printed cell separation spiral for CHO-K1 (Chinese hamster ovary) cells was developed and evaluated optically and with cell experiments. It demonstrated a high separation efficiency of over 95% at up to 20 × 10^6^ cells mL^−1^. Control over inlet and outlet flow rates allowed the operator to adjust the separation efficiency of the device while in use—thereby enabling fine control over cell concentration in the attached bioreactors. In addition, miniaturized 3D printed buffer devices were developed that can be easily attached directly to the separation unit for usage with peristaltic pumps while simultaneously almost eradicating pump pulsations. These custom pulsation dampeners were closely integrated with the separator spiral lowering the overall dead volume of the system. The entire device can be flexibly connected directly to bioreactors, allowing continuous, pulsation-free cell retention and process operation.

## 1. Introduction

Microfluidic systems in biology and biochemistry are often focused on combining several lab procedures into a single system, thereby creating a so-called lab-on-a-chip [1,2,3]. Significant attention has recently been given to adherent cell cultivation on microfluidic systems in the field of organ-on-a-chip, with the aim of furthering technological advances for medical testing and research [4,5,6,7]. The unique physical environment that is created inside microfluidic channels allows for a high degree of fluid control and facilitates interesting and frequently useful particle−fluid interactions—which can then be exploited for cell handling purposes. These interactions define the field of inertial microfluidics, which focuses on identifying and refining channel shapes and structures that transfer, mix, focus, and/or separate particles within microfluidic systems [8].

At certain flow rates, the flow inside the microfluidic channels is considered laminar (Reynolds number Re << 2000), but the flow field still falls somewhere between the Stoke state and turbulent flow. Under these circumstances, particles in motion inside a confined channel experience numerous lift and drag forces [9]. The convergent interactions of these forces result in a small number of equilibrium positions where particles tend to converge even within a straight channel—and the location of these equilibrium positions can be manipulated by changing (i.e., curving) the channel shape. These dynamics can then be exploited to finely sort or separate particles. Nawaz et al. [10] have designed a simple U-shaped channel in their hydrodynamic focusing device to achieve sub-micrometer precision in flow cytometry applications. Di Carlo et al. [11] have similarly used a sinusoidal (“snake-like”) channel to observe the self-ordering of particles and whole-blood cells inside the channel. To date, however, the most popular shape is the spiral channel—due in large part to its compact shape and stable flow field [9]. To cite one recent example, Warkiani et al. [12] successfully isolated circulating tumor cells from whole blood using a spiral microchannel, achieving a recovery rate of over 85%. In another example, Lee et al. used a spiral channel for cell cycle synchronization of human mesenchymal stem cells [13], demonstrating that an even more intricate form of sorting was possible using inertial microfluidics. Finally, in combination with the exploitation of dielectric properties, Wang et al. [14] have recently used a separation spiral to separate three different common microalgae cell types.

While most existing industrial bioprocesses are run in the fed-batch mode today, there are tremendous incentives to employ another cultivation strategy for new bioprocesses: continuous cultivation [15]. In a typical fed-batch process, cells grow and produce the protein until a maximum cell concentration is reached. Then the cellular death phase sets in, as the concentration first plateaus and then starts to actively decrease. Typically, the reactor is then harvested, and protein purification begins. With continuous cultivation, however, the cells stay inside the bioreactor and fresh medium is simply pumped in while the old medium is pumped out. As a result, using this method, the product expressed by the cells can be continuously harvested from the bioreactor [15,16]. Continuous bioprocesses thus offer the tantalizing promise of increased production flexibility and scaling, higher product quality, and lower overall cost of production when compared to fed-batch cultivations [16].

One of the primary advantages of using inertial microfluidics for cell sorting or separation is the inherent ability to facilitate precisely this sort of continuous operation while also reducing shear stresses [17]. Kwon et al. have demonstrated the usefulness of a spiral channel device for cell retention in CHO (Chinese hamster ovary) cultivation [18]. Their system boasted not only a high recovery rate, but also improved relative reliability (which is crucial for continuous use) when compared with other cell retention devices such as filter membranes.

Inertial focusing devices with multiple outlets usually operate with a fixed outlet flow rate ratio. Kwon et al. used different tubes at both outlets to manipulate fluidic resistance [18], therefore achieving an inner outlet (retentate outlet) flow rate up to 4.5 times higher than the outer outlet flow rate (permeate). However, this ratio cannot be changed during the operation of the separation device. This led to a change of flow rates at both outlets when changing the inlet flow rate—and this inflexibility prohibited the operator from regulating separation efficiency without changing the outlet flow rate, and vice versa.

In this paper, we describe a 3D printed microfluidic focusing device which can be used in both perfusion and chemostat CHO cell cultivation, wherein medium and product cells are taken continuously from the reactor in order to keep the growing cells at a given concentration. In contrast to other separation devices, by controlling the inlet and outlet flow rate with pumps, this device gives the user granular control over the number of cells taken out of the reactor—which makes it suitable for more dynamic process control and process development. To facilitate easier continuous use with peristaltic pumps, we also describe 3D printed buffer devices which minimize the pulsations created by the pump, and can be easily integrated with the separation spiral, leading to small system volume. The design presented here includes threaded ports commonly used in HPLC (high-performance liquid chromatography) applications, which create a secure connection with well-known, off-the-shelf parts. Additionally, the 3D printed designs can also be easily changed in CAD (computer-aided design) software or integrated into other systems to adapt to new processes.

## 2. Materials and Methods

### 2.1. Fabrication of Microfluidic Devices

All CAD models were designed in Solidworks 2020 (Dassault Systèmes, Vélizy-Villacoublay, France), and then 3D printed using the Projet 2500 plus MultiJet 3D printer (3D Systems, Rock Hill, SC, USA). The printer has a resolution of 800 × 900 DPI in x and y directions and a layer height (z direction) of 32 µm. The surface roughness (arithmetic average of roughness) of the printed parts were 9, 9 and 2 µm (xyz) [19]. The printing material used was M2S-HT90 (3D Systems), which forms solid polyacrylate after being exposed to UV rays during the printing process. The support material used was M2-SUP (3D Systems). After completion of the print, the parts were cooled at −18 °C in a freezer for approximately 10 min, and then separated from the print platform. The printed parts were then placed in a hot water vapor bath (to melt the wax-like support material) for approximately 20 min. Afterwards, the parts were placed in a heated oil bath (paraffin oil at 65 °C), and the channels were then flushed with warm oil and submerged in an ultrasonic bath with paraffin oil in order to dissolve any remaining residual support material. Finally, any potential lingering oil residues were removed using warm soap water (Fairy Ultra detergent, Procter & Gamble, Cincinnati, OH, USA) in yet another ultrasonic bath.

### 2.2. Separation Device Design

The separation spiral was developed using design guidelines and the design equation articulated by Di Carlo [8] to facilitate focusing of 18 µm particles (or cells) at a flow rate of 1000 µL min^−1^ (see Equation (1)). Additionally, the height of the spiral channel was 200 µm, since prior experiments have demonstrated that this size can be reliably produced using the 3D printer [19,20].

(1) $$Lf=\frac{\pi \mu H^{2}}{\rho U_{m}a^{2}f_{L}}$$

**Figure 1:**
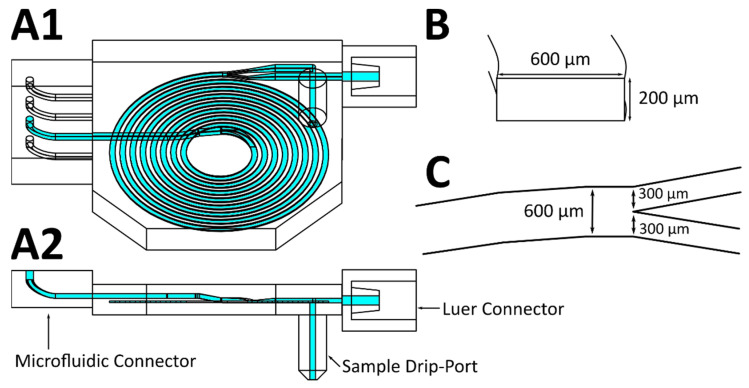
CAD drawing of the spiral separator. (**A1**) Separator design used for cell retention testing (**A2**) side view of the design. Dolomite Microfluidic Connector (Dolomite Microfluidics, UK) on the left, slip-on luer connector for syringes on the right, sample collection port on the bottom. (**B**) Cut view of the spiral channel with a height of 200 µm and width of 600 µm. (**C**) Top view of the split at the end of the spiral channel into two 300 µm wide outlet channels.

Lf is the channel length required for focusing to equilibrium positions, μ is the viscosity of the fluid (water; 0.001 kg M^−1^ s^−1^), H is the long side of the channel (600 µm width), ρ is the fluid density (1000 Kg m^−3^), $U_{m}$ is the maximum velocity (0.19375 m s^−1^, calculated with a flow rate of 930 µL min^−1^ in a 200 µm by 600 µm channel multiplied by 1.5 to estimate the maximum velocity in the channel center [8]), a is the diameter of the particles or cells (18 µm [21]), and $f_{L}$ is the lift factor (0.04 estimate from Di Carlo [8]) 8]. The calculated separation channel is 450 mm long, 200 µm deep, and 600 µm wide. Figure 1 shows the resulting CAD (computer aided design) model of the separation spiral developed to focus CHO-K1 cells while offering control over the separation efficiency due to a second pump connected to one spiral outlet.

For initial experiments using fluorescent particles, the spiral channel used was 830 mm long, in order to compensate for foreseeable disturbances due to surface roughness. Rough edges on the channel wall could lead to disturbances of the laminar flow, therefore pushing the particles away from the equilibrium position. For the cell experiments, a length of 372 mm was chosen, based on the results obtained in previous experiments. A second outlet was also added to the design (see Figure 1) in order to allow for different flow rates and the separation of cells, thereby creating two outlets with a width of 300 µm each. In experiments with pulsation buffers, a second inlet was added 1.7 mm in front of the end of the spiral; this inlet was used to add ink solution to the stream, in order to facilitate easier and more accurate observation of flow pulsations.

### 2.3. Buffer Device Design

Two different buffer devices were designed to eliminate flow pulsation by the peristaltic pumps. These devices were based on a buffer design idea generated by Kang et al., wherein needles were inserted into plastic syringes to act as pulsation buffers and bubble traps [22]. This buffer design was modified to be more compact and integrated, with added PTFE tube connectors for more rigid connections. The inlet pump (Ismatec REGLO Digital, Cole-Palmer GmbH, Wertheim, Germany) was connected to a smaller 1 mL buffer device, while the outlet pump was connected to a larger 5 mL buffer device. The buffer devices were glued to the separator device using acrylate glue (UHU Sekundenkleber, Bolton Adhesives, Rotterdam, Netherlands).

### 2.4. Experimental Setup

In a first experiment, fluorescent particles with a size similar to CHO cells (CHO cell size approximately 18 µm [21], Fluoresbrite YG Microspheres 20.0 µm; Polysciences, Warrington, PA, USA) were pumped through the spiral separator using a syringe pump (AL-1000, World Precision Instruments, Sarasota, FL, USA) at 1000 µL min^−1^. Four different concentrations were used: 0.5, 1, 2, and 5.56 (stock concentration) × 10^6^ particles per mL. The flow was evaluated microscopically (Olympus IX-50, Olympus Corporation, Tokyo, Japan).

To evaluate the performance of the separator that was designed to isolate CHO cells, four different cell concentrations (CHO-K1) were tested: 5, 10, 15, and 20 × 10^6^ cells per milliliter. Additionally, four different inlet flow rates were also tested to determine separation efficiency at different flow rates: 500, 700, 1000, and 1300 µL min^−1^. Another factor that influences separation efficiency is the outlet ratio—and so to test relative outlet ratios, a syringe pump was connected to one outlet drawing with a set flow rate and eight different outlet ratios were deployed with each inlet flow rate. The syringe pump connected to the inlet was placed upright above a magnetic stirrer, with a stir bar placed inside the syringe to keep the cell solution homogenized during the experiment. The second syringe pump was connected directly to the separation spiral outlet. After each change in outlet flow rate, 250 µL of cell suspension were discarded before collecting the sample to allow the flow inside the spiral to stabilize.

### 2.5. CHO Cell Cultivation and Preparation

CHO-K1 cells were cultivated in CHO-TF cell culture medium (Xell AG, Nordrhein-Westfalen, Germany) in 125 mL, 250 mL, or 500 mL baffled Erlenmeyer shake flasks with vent caps (Fisher Scientific GmbH, Schwerte, Germany) at 37 °C and 5% CO_2_ in humidified atmosphere on a shaker (MaxQ CO_2_ Plus, Thermo Fisher Scientific Inc., Bartlesville, OK, USA) at 120 rpm. Each passage was started with a concentration of 0.4 × 10^6^ cells mL^−1^, and cells were harvested at a concentration of 10 × 10^6^ cells mL^−1^. If needed, the cells were first centrifuged and then resuspended to achieve higher concentrations. Following resuspension, the cells were shaken gently at 120 rpm in 50 mL reaction tubes (Fisher Scientific GmbH, Germany) for 1 h, in order to disperse any cell aggregates that might have coalesced before the experiment began.

## 3. Results and Discussion

### 3.1. Inertial Focusing Using the Spiral Separator

**Figure 2:**
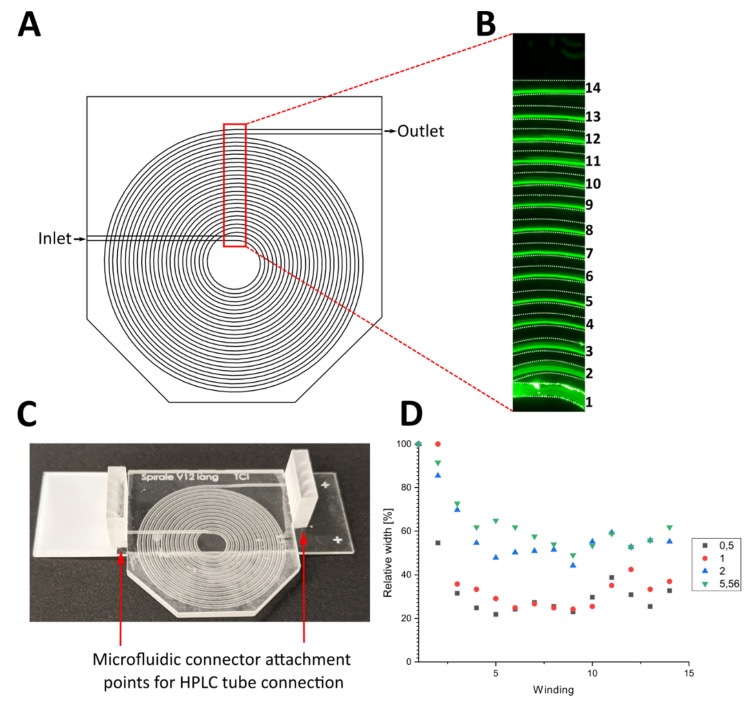
(**A**) Top view of the CAD drawing of the separator spiral. (**B**) Image of the fluorescent particle flow in the red region of A. Channel walls were highlighted with white dotted lines. Numbers on the right represent the number of the spiral winding. (**C**) Photo of the 3D printed spiral separator with microfluidic connector attachment points on the inlet and outlet. (**D**) Relative width of the fluorescent particle flow in relation to the channel width after each winding of the spiral at particle concentrations of 0.5, 1, 2 and 5.56 × 10^6^ particles per mL.

To provide direct insight into the focusing mechanics of the spiral separator, fluorescent particles were pumped through the 3D printed device—at different flow rates, and in different concentrations—using a syringe pump. The flow of particles was observed under a fluorescence microscope at the design flow rate of 1000 µL min^−1^ (see Figure 2). The spiral channel used in this experiment (see Figure 2A) was elongated to 830 mm in order to compensate for foreseeable eventual negative effects arising from the irregular print surfaces increasing the focusing distance. Figure 2B shows a microscopic image of 0.5 × 10^6^ particles per mL. To determine the position of the focused particle stream inside the channel, the fluorescence images were analyzed using ImageJ by determining the width of the channel and the width of the fluorescent stream. The number of bright pixels (higher than the brightness threshold of 71) along a vertical line was taken as the stream width. A graphical representation of the analysis is shown in the Supplementary Information (Figure S1). Figure 2C shows the printed separation spiral and Figure 2D the relative width of the particle stream inside the channel at each winding for the four different particle concentrations.

The particles initially flowed across the whole width of the channel (100%) at the spiral inlet, but they were observed to quickly focus near the inner channel wall. Lower particle concentrations of 0.5 and 1 × 10^6^ particles per mL revealed a sharper focused stream, with a relative width of 31 and 35% at the third winding, respectively. Higher concentrations of 2 and 5.5 × 10^6^ particles per mL resulted in broader streams, followed by a subsequent focusing with the smallest width of 50–60% after seven windings. Since the position of the particle flow did not change significantly after the seventh winding (372 mm channel length) of the spiral channel and the back pressure increases with the channel length, subsequent experiments were conducted using a shorter channel length of 372 mm. Since the fluorescent particles used in this experiment do not represent the different sizes of CHO cells expected in a real cultivation, where the cell size changes depending on the growth phase of the cell, the results are only a first proof of concept. The separation efficiency of the device has to be evaluated using real cells.

### 3.2. Focusing of CHO-K1 Cells

**Figure 3:**
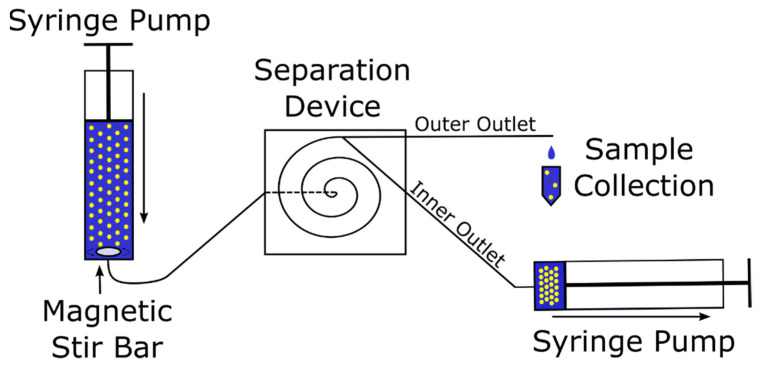
Schematic of the experimental setup to evaluate the separator performance. The inlet syringe pump is placed upright above a magnetic stirrer and a magnetic stir bar is placed inside the syringe to mix the cell solution during the experiment.

To evaluate the performance of the spiral separator, CHO-K1 cell solutions with four different concentrations were pumped through the separation device using a syringe pump. To manipulate the flow ratio of both outlet channels at the end of the spiral to control the separation efficiency, a second syringe pump was also deployed to withdraw from the inner outlet at eight different flow rates per inlet flow rate. The samples were taken from the outer outlet by collecting the droplets using a 1.5 mL collection tube. Subsequently, the cell concentration was analyzed using CEDEX HiRes. Figure 3 illustrates the experimental setup.

The cell concentration was multiplied by the flow rate to determine the cell loss rate, i.e., the number of cells being pumped out of the outer outlet per minute. This can be compared to a cell growth rate when the system is used with a bioreactor, in order to control the cell concentration inside the reactor. Additionally, the efficiency of the separation device was calculated by dividing the cell loss rate by the theoretical cell loss rate at the outlet flow rate without any separation effects (see Equation (2)).

(2) $$efficiency\;\left(\%\right)=100-\left(\frac{cell\;loss\;rate\;}{OO\;Flow\;Rate\times input\;cell\;conc.}\right)$$

where OO Flow Rate is the flow rate at the outer outlet and input cell conc. is the cell concentration pumped into the separation device.

**Figure 4:**
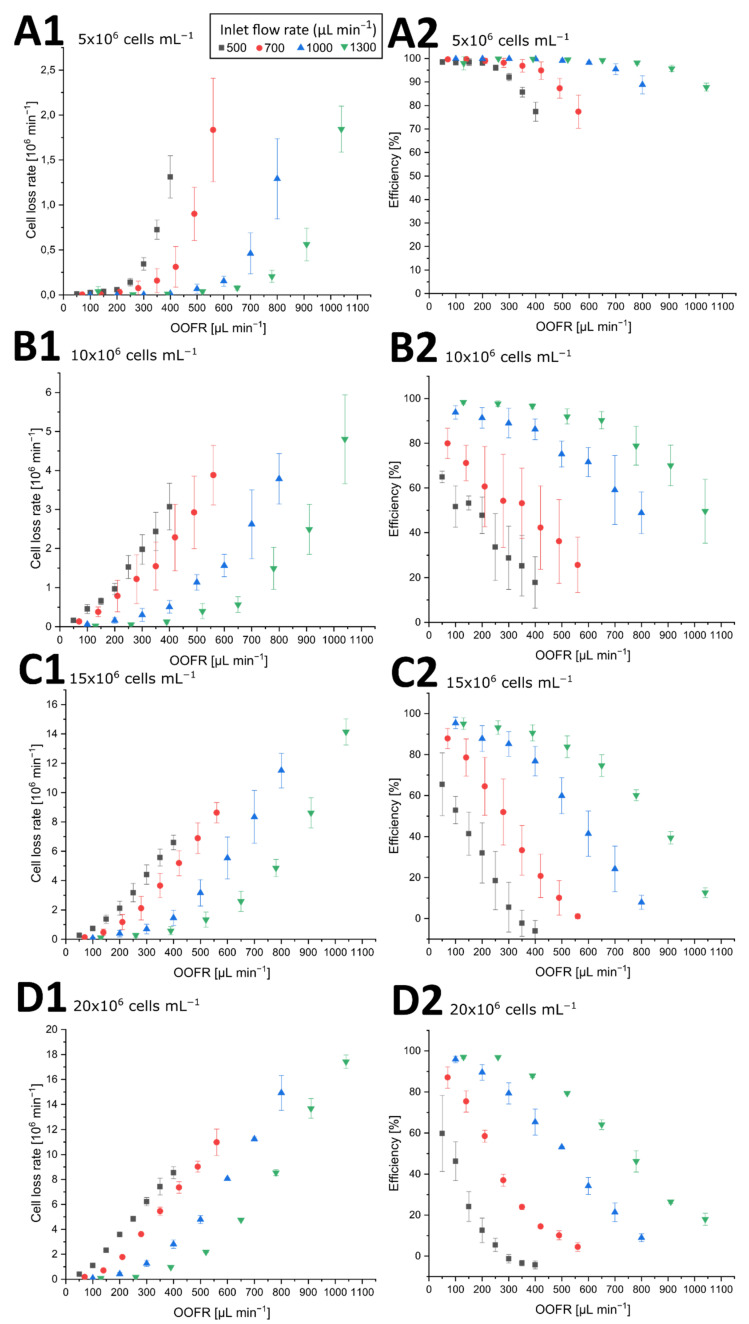
Cell loss rate and efficiency of the cell retention device at four different cell concentrations. Blue markers: inlet flow rate of 500 µL min^−1^; orange markers: 700 µL min^−1^; grey markers: 1000 µL min^−1^; yellow markers: 1300 µL min^−1^. Error bars represent the standard deviation of three biological replications. (**A1**) Cell loss rate (× 10^6^ cells min^−1^) as a function of the outer outlet flow rate (OOFR; mL min^−1^) at different inlet flow rates with an inlet cell concentration of 5× 10^6^ cells mL^−1^. (**A2**) Separation efficiency as a function of the OOFR at different inlet flow rates with an inlet cell concentration of 5 × 10^6^ cells mL^−1^. (**B1**) Cell loss rate (× 10^6^ cells min^−1^) as a function of the OOFR at different inlet flow rates with an inlet cell concentration of 10 × 10^6^ cells mL^−1^. (**B2**) Separation efficiency as a function of the OOFR at different inlet flow rates with an inlet cell concentration of 10 × 10^6^ cells mL^−1^. (**C1**) Cell loss rate (× 10^6^ cells min^−1^) as a function of the OOFR at different inlet flow rates with an inlet cell concentration of 15 × 10^6^ cells mL^−1^. (**C2**) Separation efficiency as a function of the OOFR at different inlet flow rates with an inlet cell concentration of 15 × 10^6^ cells mL^−1^. (**D1**) Cell loss rate (× 10^6^ cells min^−1^) as a function of the OOFR at different inlet flow rates with an inlet cell concentration of 20 × 10^6^ cells mL^−1^. (**D2**) Separation efficiency as a function of the OOFR at different inlet flow rates with an inlet cell concentration of 20 × 10^6^ cells mL^−1^.

Figure 4 shows the cell loss rate and the efficiency of three biological replications as a function of the outer outlet flow rate at different input flow rates for four different cell concentrations.

At the concentration of 5 × 10^6^ cells mL^−1^ (see Figure 4(A1,A2)), all inlet flow rates depicted a hockey stick curve. While at the lowest outer outlet flow rates (OOFR) cell loss rates were minimal, the cell loss rate increased at an outlet flow rate approximately half the inlet flow rate (0.14 × 10^6^ cells min^−1^ at 250 µL min^−1^ OOFR at 500 µL min^−1^ inlet flow rate; 0.15 × 10^6^ cells min^−1^ at 350 µL min^−1^ OOFR at 700 µL min^−1^ inlet flow rate; 0.06 × 10^6^ cells min^−1^ at 500 µL min^−1^ OOFR at 1000 µL min^−1^ inlet flow rate; 0.08 × 10^6^ cells min^−1^ at 650 µL min^−1^ OOFR at 1300 µL min^−1^ inlet flow rate). Consequently, the efficiency of the separator declined at higher OOFRs, with efficiencies well over the 95% observed at low OOFRs of 100–200 µL min^−1^, but dropping to 77% at an inlet flow rate of 500 µL min^−1^ at OOFR of 400 µL min^−1^. Higher inlet flow rates showed higher separation efficiencies even at greater OOFRs, with 87% efficiency at an OOFR of 1040 µL min^−1^. Lower retention efficiencies led to greater standard deviations, with a maximum of 7.08% at an OOFR of 560 µL min^−1^ at an inlet flow rate of 700 µL min^−1^. Similarly, the standard deviation of the cell loss rate increased with higher cell loss rates.

At a cell concentration of 10 × 10^6^ cells mL^−1^ (see Figure 4(B1,B2)), the cell loss rate increased at lower outlet flow ratios and a stronger dependency of the inlet flow rate was observed. At an inlet flow rate of 500 µL min^−1^, the lowest cell loss rate of 0.32 × 10^6^ cells/min was at 50 µL min^−1^ OOFR. The cell loss rate then increased almost linearly with a higher OOFR to 3.07 × 10^6^ cells min^−1^ at 400 µL min^−1^. The increase of cell loss rate was similar at 700 µL min^−1^ inlet flow rate. However, at the 1000 µL min^−1^ inlet flow rate, the increase of the cell loss rate with OOFR was more nearly exponential—much like the increase at a cell concentration of 5 × 10^6^ mL^−1^ with a loss rate of 0.4 × 10^6^ cells mL^−1^ at OOFR of 400 µL min^−1^. Similar results were also observed with the 1300 µL min^−1^ inlet flow rate. Consequently, the separation efficiency at lower inlet flow rates was significantly lower at 10 × 10^6^ cells mL^−1^. However, at higher OOFRs, the efficiency dropped to 50% at both inlet flow rates of 500 and 700 µL min^−1^. Higher input flow rates also provided higher separation efficiencies at relatively high OOFRs, again like the separation efficiency at 5 × 10^6^ cells mL^−1^. The standard deviations of the cell loss rate increased to a maximum of 1.3 × 10^6^ cells min^−1^ at an inlet flow rate of 1300 µL min^−1^ with the cell loss rate, while the standard deviation of the efficiency was generally greater (maximum of 18%) than the deviations at an input cell concentration of 5 × 10^6^ cells mL^−1^.

At a cell concentration of 15 × 10^6^ cells mL^−1^ (see Figure 4(C1,C2)), the effectiveness of the separation device at lower input flow rates decreased at lower OOFRs. The cell loss rates at input flow rates of 500 and 700 µL min^−1^ grew almost linearly with the OOFR. At both the 500 and 700 µL min^−1^ input flow rate, the effectiveness reached 0% and below 0% at the highest OOFRs. The negative effectiveness could potentially be explained by cells being repelled from the inner channel wall into the middle of the channel, therefore increasing the concentration at higher OOFRs, while the calculated theoretical cell loss rate assumed an even cell distribution in the channel. Higher input flow rates resulted in higher effectiveness at lower OOFRs, but also resulted in greater decreases in effectiveness at OOFR over 500 µL min^−1^. The standard deviation of the cell loss rate increased slightly with the cell loss rate to a maximum of 1.79 × 10^6^ cells min^−1^ at an inlet flow rate of 1000 µL min^−1^. The greatest standard deviation of the separation efficiency was 15%, while the largest deviation of the cell loss rate was 1.8 × 10^6^ cells min^−1^.

At a concentration of 20 × 10^6^ cells mL^−1^ (see Figure 4(D1,D2)) the cell loss rate almost increased linearly at lower input flow rates, but the increase was also steeper at higher input flow rates. The efficiency of the separation device dropped at lower OOFRs. At 500 µL min^−1^ input flow rate, the highest efficiency was 60% at 50 µL min^−1^ OOFR and dropped to just 5% at 250 µL min^−1^ OOFR. This resulted in the cell loss rate rising linearly with OOFR at 500 µL min^−1^. Higher input flow rates showed a similar drop in efficiency as at 15 × 10^6^ cells min^−1^. Only at 1300 µL min^−1^ input flow rate was the efficiency still over 95% at an outlet flow rate of 260 µL min^−1^, which was also visible in the exponential increase of the cell loss rate with higher OOFR. Standard deviations were generally lower than at other inlet flow rates, with a maximum deviation of the efficiency of 18.5%.

In conclusion, the separation efficiency of the separator device could demonstrably be controlled by manipulating the inlet and/or outlet flow rate. This enabled the device to either be operated in a most effective state with almost complete cell retention (over 95% at 20 × 10^6^ cells min^−1^), or set to achieve a specific cell loss rate in order to facilitate steady cell concentrations within the bioreactor. However, the standard deviations of the biological replications were generally much greater than the instrument error (5%) of the CEDEX. HiRes. Therefore, when using this system for cell concentration control in a bioreactor, the cell concentration has to be monitored to adjust the inlet and outlet flow rate to achieve the desired retention efficiency, especially when a higher cell loss rate and a lower retention efficiency is required.

**Figure 5:**
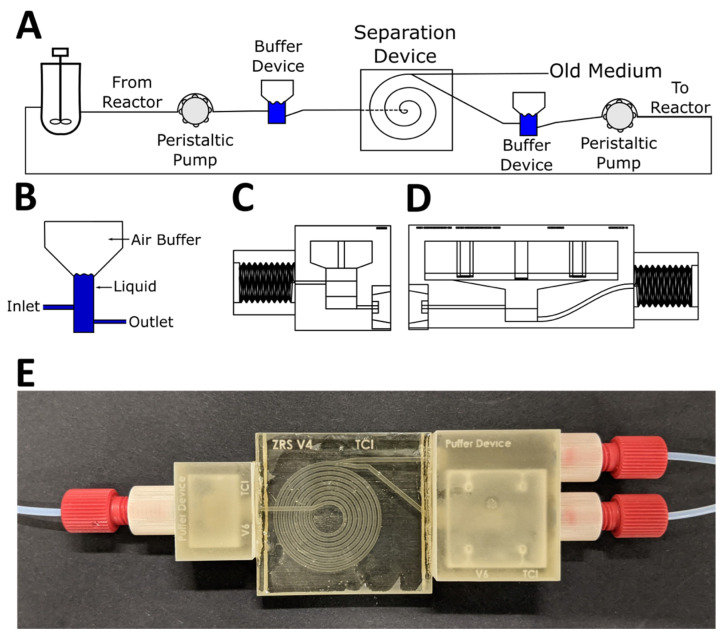
(**A**) Example of an experimental setup with peristaltic pumps, buffer devices, and the cell separation device for use with a bioreactor. (**B**) Schematic side view of a buffer device, where the bottom part fills up with liquid while the trapped air works as a damper. (**C**) Side view of the smaller 1 mL buffer used behind the inlet pump with 1 mL air volume; flangeless fitting connector on the left, glue connection on the right side of the device. (**D**) Larger buffer device with a 5 mL air buffer used in front of the outlet pump; flangeless fitting connector at the right and glue connector at the left side of the device. Additional CAD views and dimensions are available in the Supplementary Information (Figure S2). (**E**) Top view of the 3D printed buffer devices and spiral separator fitted with PTFE tubes and flangeless fittings. The outer outlet channel (see “Old Medium” in (**A**)) is parallel to the outlet buffer device and not connected to the buffer itself. The spiral device was coated with nail polish to improve the translucency.

The viability of the cells was determined using trypan blue staining in CEDEX HiRes. The cell viability after being pumped through the separation device was generally very similar to the viability prior to separation (above 98%). The viability of the very low cell concentration (“Old Medium” in the operational setup in Figure 5A) only began to decrease at very high separation efficiency rates (> 95%). This might be an artifact of the low cell concentration (1 × 10^6^ cells min^−1^ and lower). Additionally, it is worth noting that these cells are discarded in the experimental setup. In an additional experiment with cells being pumped through the separation device and then cultivated for 48 h, trypan blue staining and LDH assays were performed. Cell viability was revealed to be 97.6% after 48 h with LDH activity of 9.8 U mL^−1^. The reference culture showed similar values, with a viability of 98.8% and LDH activity of 36.8 U mL^−1^.

### 3.3. Buffer Devices to Eliminate Pulsation

The separation device described above was designed to be used in perfusion culture systems. This necessitates constant flow, in order to reduce the time cells spend in the separation system and tubing from and to the system and also to prevent cells from settling in the system and tubing. While in the separation experiments syringe pumps were used for their flow precision and low pulsation, these pumps cannot be used to achieve constant pumping. Therefore, peristaltic pumps were also explored.

Peristaltic pumps inherently create flow pulsations, due to their working principle. The previous results demonstrate that variations in flow rates have a great influence on separation efficiency. Accordingly, in an effort to lessen the pulsation effects created by peristaltic pumps, buffer devices—based on the idea of using plastic syringes as buffers and bubble traps described by Kang et al. [22]—were designed and printed. Similar pulsation dampeners are also available commercially (e.g., MasterFlex Pulse Dampener by Cole-Parmer, USA), but regrettably these lack size adaptability and are too large for use within microfluidic systems. Additionally, tubing is required to connect the devices, which increases the dead volume of the system and therefore the time spent in the system by CHO cells, where oxygen and nutrient supply is limited.

The air trapped inside the buffer volume of these buffer devices can be used to dampen the pulses of the peristaltic pumps: the air becomes pressurized when the buffer device is placed in front of the inlet of the separation spiral, because of the higher pressure in the spiral, and when placed behind the outlet of the spiral and in front of the second pump, the pump draws air out of the device and relative negative pressure is created inside the buffer device (which has the same dampening effect). The buffer devices used in this experiment were printed separately and glued to the spiral separator system, in order to keep it compact and limit the tubing required. The buffer devices and the resulting experimental setup is shown in Figure 5.

**Figure 6:**
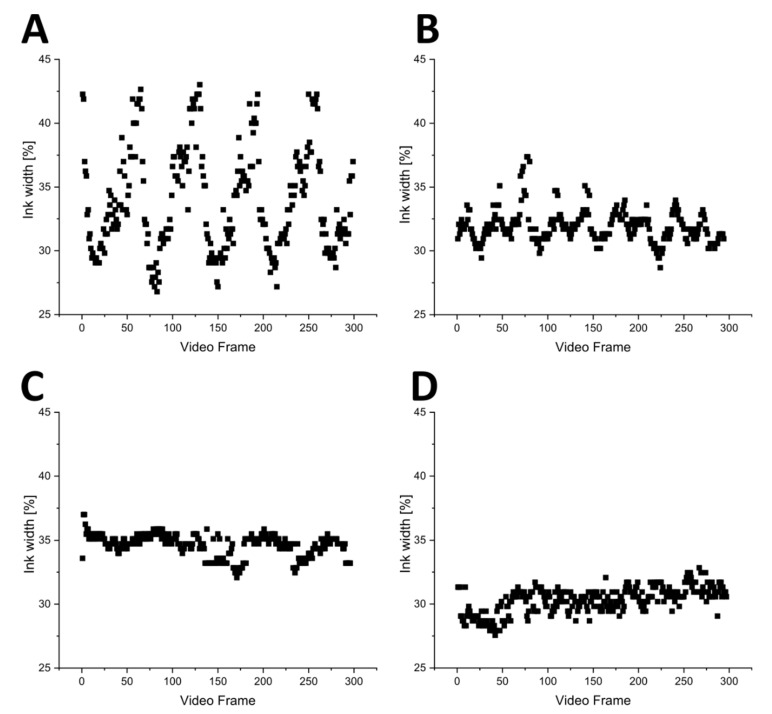
(**A**) Ink width (%) in different video frames without pulsation devices. (**B**) Ink width (%) in different video frames with the outlet pulsation device attached. (**C**) Ink width (%) in different video frames with a pulsation device at the inlet and the outlet, respectively. (**D**) Ink width (%) in different video frames with syringe pumps for reference.

The effectiveness of these buffer devices was determined microscopically, by observing the flow of blue ink. An additional channel at the end of the spiral was used to add ink solution with a syringe pump, while water was pumped through the separation device. Twenty-second-long videos of the pulsating flow were then analyzed by determining the pixel brightness of the cross section at the end of the spiral of every frame in ImageJ. A brightness threshold of 163 was used to determine the width of the ink-filled area of the channel. A graphical representation of the analysis is shown in the Supplementary Information (Figure S3). The results are shown in Figure 6.

The results clearly illustrate the regular pulsation of the peristaltic pumps with the ink width pulsing between 27 and 44% without any pulsation devices (Figure 6A). With one pulsation device connected in front of the outlet peristaltic pump, the pulsation intensity was visibly minimized and the ink width pulses fell to between 29 and 37% (Figure 6B). When both pulsation devices were connected (one behind the inlet pump and the second in front of the outlet pump), no clear periodic pulses were visible in the data (see Figure 6C), and the ink width fell between 32 and 37%. Similarly, the reference experiment using syringe pumps instead of peristaltic pumps showed no clear pulsation, and the ink width similarly fell between 27 and 33% (Figure 6D). On the basis of these results, we conclude that the buffer devices were able to significantly reduce the pulsation induced by the peristaltic pumps. The slight difference in overall ink width between Figure 6C,D can perhaps be explained by a variation in flow rate, since the actual flow rate of the peristaltic pumps differs more from the set flow rate than the flow rate of syringe pumps.

Of particular note is the fact that the complete setup with buffer devices and separator spiral described herein did not leak even during extended use (up to 12 days with a constant flow rate of 1 mL min^−1^)—while the aforementioned syringe design by Kang et al. [22] proved to be very unreliable, leaking from movement of the tubing and/or material wear after just a matter of hours. Additionally, by using acrylate glue (super glue) for assembly, the assembly of the system described herein requires only a few minutes. Due to the customized small buffer devices and direct connection of buffer devices and separator spiral, the dead volume within this system was calculated to be only 1.59 mL (in CAD software, considering the inlet buffer is filled completely, while only the bottom of the outlet device is filled, as observed).

## 4. Conclusions

This study presents a 3D printed cell separation device with optional additional 3D printed pulsation devices for use with peristaltic pumps. The experimental setup described herein allows the user to control the inlet and outlet flow rate in order to adjust the separation efficiency of the system during operation. The separation efficiency and cell loss rate were determined at four different cell concentrations (5, 10, 15, and 20 × 10^6^ cells mL^−1^), and at inlet flow rates of both 500 to 1300 µL min^−1^. Higher inlet flow rates led to an increased separation efficiency of over 95%, even at up to 20 × 10^6^ cells mL^−1^ at outlet flow rates of up to 260 µL min^−1^. The separation device did not evidence any significant negative effect on cell viability. Additionally, pulsation buffer devices were also developed to enable the usage of peristaltic pumps within a continuous bioreactor setup. Effective dampening of the inherent pulsation created by peristaltic pumps was also successfully demonstrated, with results achieved that are comparable to those seen in systems using syringe pumps. All 3D printed parts can be easily connected, and allow for reliable connections to PTFE tubing with flangeless fittings. The complete device did not develop leaks even after 12 days of use, while the integration of dampening devices reduced the tube connections needed. Due to the use of 3D printing, modification of this design is readily achievable (e.g., for use at larger bioreactors or different tubing fixtures). In future experiments, the reliability of the system and controllability of the separation efficiency for longer cultivation periods up to multiple weeks must be proven. Then, this device or a similar design might allow the operator to set specific cell concentrations in perfusion culture cultivations, thereby optimizing the cell concentration to increase product yield more precisely. In additional experiments, the bioprocess can be optimized by elongating the protein production phase due to cell retention and the control of the cell concentration in the bioreactor provided by this separation device.

## Data Availability

The data presented in this study are openly available in Research Data Repository of the Leibniz University of Hannover at https://doi.org/10.25835/0098015 (accessed on 3 June 2021).

## Supplementary Materials

The following are available online at https://www.mdpi.com/article/10.3390/mi12091060/s1, Figure S1: Analysis of the fluorescence microscope images, Figure S2: CAD views and inner dimensions of the 1 mL and 5 mL pulsation dampeners, Figure S3: Analysis of the microscopic images of the pulsation experiments, CAD files of pulsation dampeners.
