# Supplementary material for: 3D Printed Microfluidic Spiral Separation Device for Continuous, Pulsation-Free and Controllable CHO Cell Retention
Source: Micromachines (Basel). 2021 Aug 31;12(9):1060. doi: 10.3390/mi12091060 (PMC8470376; doi:10.3390/mi12091060)
Supplement: Supplementary file 1 [file micromachines-12-01060-s001.zip › Supplementary Information.pdf]

Supplementary Information

3D Printed Microfluidic Spiral Separation Device for continuous, pulsation-free and controllable CHO Cell Retention

Anton Enders , John-Alexander Preuß and Janina Bahnemann

Figure S1 shows the analysis of the microscopic images of fluorescent beads in section 3.1.

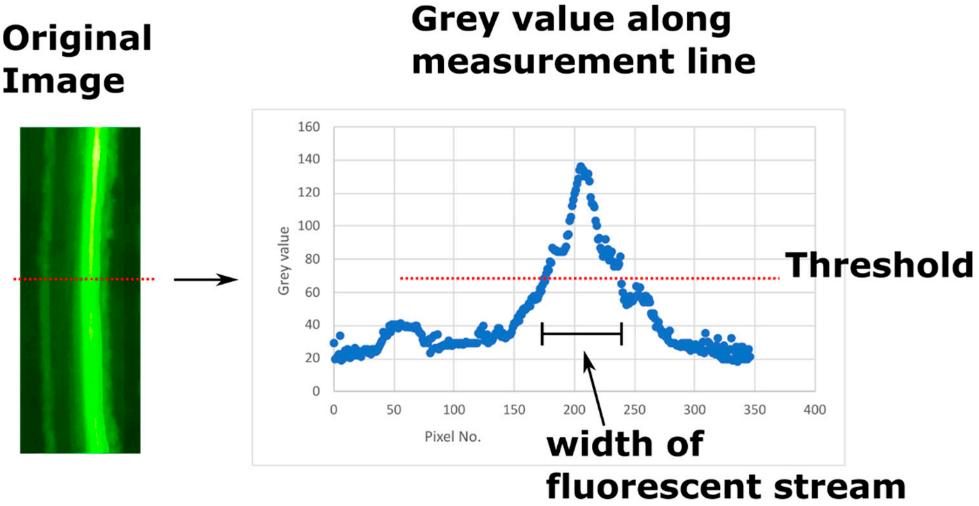

Figure S1: Analysis of the fluorescence microscope images. The analysis was performed in ImageJ by analysing the grey value (brightness) of each pixel along a measurement line. Grey values above a threshold of 71 were identified as bright fluorescent, which was the basis to determine the width of the fluorescent stream.

Figure S2 shows different views of the pulsation dampeners used in section 3.3.

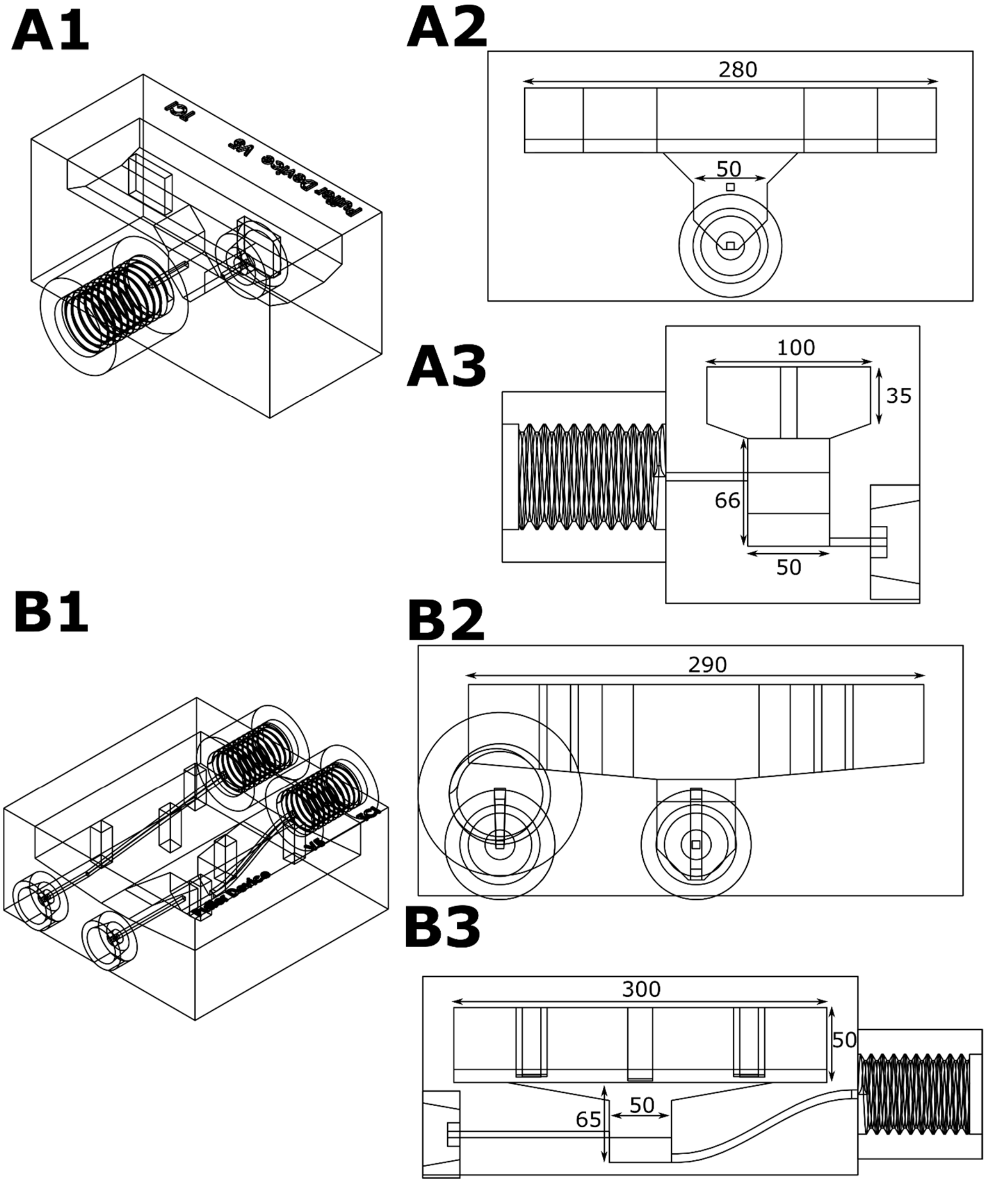

Figure S2: CAD views and inner dimensions of the 1 mL and 5 mL pulsation dampeners. All the dimensions are in micrometers (µm). A1: 3D view of the 1 mL inlet pulsation dampener. To enhance structural integrity of the device, two columns are printed in the middle of the cavity. A2: Front view of the 1 mL dampener. A3: Side view of the dampener. B1: 3D view of the 5 mL outlet pulsation dampener. To enhance structural integrity of the device, six columns are printed in the cavity. B2: Front view of the dampener. B3: Side view of the dampener.

Figure S3 shows the analysis of the pulsation experiments from section 3.3.

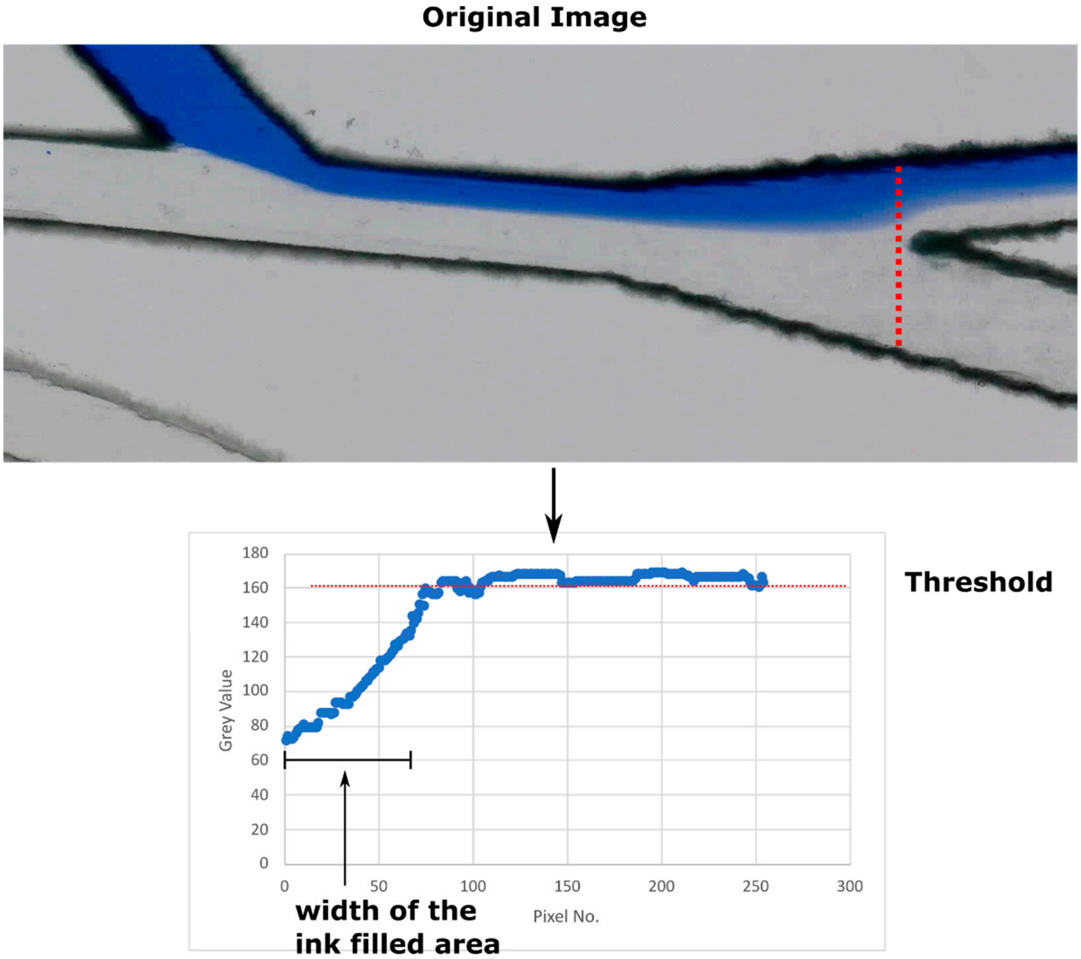

Figure S3: Analysis of the microscopic images of the pulsation experiments. Each frame of the 20 second long videos was analyzed using ImageJ by determining the grey value (brightness) of each pixel along the measurement line. A theshold of 160 was chosen to determine the bright part of the channel, while darker pixels indicated the ink filled part. Therefore, the width of the ink filled part was measured.
